# Use of platelet rich plasma for skin rejuvenation

**DOI:** 10.1111/srt.13714

**Published:** 2024-04-22

**Authors:** Lam Kar Wai Phoebe, Kar Wai Alvin Lee, Lisa Kwin Wah Chan, Lee Cheuk Hung, Raymond Wu, Sky Wong, Jovian Wan, Kyu‐Ho Yi

**Affiliations:** ^1^ Perfect Skin Solution Hong Kong Hong Kong; ^2^ EverKeen Medical Centre Hong Kong Hong Kong; ^3^ Asia‐Pacific Aesthetic Academy Hong Kong Hong Kong; ^4^ Leciel Medical Centre Hong Kong Hong Kong; ^5^ Division in Anatomy and Developmental Biology Department of Oral Biology Human Identification Research Institute BK21 FOUR Project Yonsei University College of Dentistry Seoul South Korea; ^6^ Maylin Clinic (Apgujeong) Seoul South Korea

**Keywords:** cell‐ and tissue‐based therapy, platelet‐rich plasma, rejuvenation, skin aging, wrinkling

## Abstract

**Objective:**

Platelet‐rich plasma (PRP) is recognized as a safe and effective therapy for regenerative skin healing and rejuvenation, utilizing autologous blood enriched with various growth factors. This review aims to assess the efficacy of PRP treatments for skin rejuvenation.

**Methods:**

Keywords such as “platelet‐rich plasma,” “rejuvenation,” “skin aging,” and “wrinkles” were queried on Ovid, PubMed, and MEDLINE to identify pertinent studies on PRP treatment for skin rejuvenation.

**Results:**

Analysis revealed that PRP treatment led to significant enhancements in multiple facial parameters after one to three sessions. Improvements were noted in skin pore size, texture, wrinkle reduction, pigmented spots, collagen density, hyaluronic acid levels, and protection against ultraviolet damage. Combining PRP with hyaluronic acid demonstrated a synergistic effect, particularly enhancing skin elasticity in patients with lower body mass index and firmness in individuals aged 50s and 60s. Incorporating both physical and biometric data for assessment proved superior to relying solely on physical observations for evaluating subtle skin quality and structural changes.

**Conclusion:**

This study underscores the efficacy of PRP monotherapy for skin rejuvenation and emphasizes the necessity of standardizing PRP preparation protocols in future investigations. Heightened awareness and advancements in technology have contributed to the emergence of higher‐quality, less biased studies supporting PRP as a reliable and safe therapeutic option for skin rejuvenation.

## 1 INTRODUCTION

Skin aging is a complex process that affects all skin layers, resulting in changes in texture, tone, and elasticity. Environmental factors such as oxidative stress, pollution, and UV radiation exposure can accelerate skin aging and illness.^1^

Platelet‐rich plasma (PRP) is an autologous biological product that involves the injection of activated platelets, which stimulate the release of growth factors, triggering fibroblast proliferation and healing via the formation of new collagen, elastin, and extracellular matrices. Two critical aspects that can influence treatment outcomes are the patient's factors and the PRP preparation method.^2^ Pro‐inflammatory cytokines were shown to be higher in PRP obtained from irritated skin, potentially counteracting the anti‐inflammatory effects of PRP.^3^ On the other hand, age may alter the blood composition (e.g., platelet counts), which affects the levels of growth factors in PRP. Therefore, it is important to select suitable patients, evaluate skin conditions, past medical history, and current drug use before performing PRP procedures to ensure optimal results.

PRP was first introduced by hematologists in the 1970s to describe plasma with a higher platelet count than peripheral blood.^4^ Initially employed in transfusions for patients with thrombocytopenia, PRP quickly gained recognition as a promising therapeutic tool, with applications across various medical specialties.^5^

A decade later, PRP found its place in maxillofacial surgery, labelled as platelet‐rich fibrin (PRF) because of its fibrin content. The anti‐inflammatory properties of PRP, combined with its ability to promote cell proliferation, proved to be invaluable in various surgical applications.^6^ In subsequent years, PRP gained considerable traction in orthopedic surgery, particularly in the treatment of sports‐related injuries, where it became widely adopted and acclaimed.^7^ Beyond orthopedics, PRP has been utilized in diverse specialties, including cardiac surgery, pediatric surgery, gynecology, plastic surgery, ophthalmology, and urology.^8^

Since the early 2010s, PRP has emerged as a potential treatment for urologic diseases and sexual health, such as erectile dysfunction and female sexual dysfunction. Initial studies in animal models have yielded promising results, indicating improvements in nerve regeneration and erectile function.^9^, ^10^ Additionally, PRP is under investigation as a treatment option for female stress urinary incontinence.^11^ Human studies, particularly in the United States, have assessed the safety and feasibility of PRP injections for erectile dysfunction and female sexual dysfunction, reporting positive outcomes reported.^11^, ^12^ further research is necessary to comprehensively understand the efficacy and long‐term effects of PRP in treating both erectile and female sexual dysfunction.

More recently, there has been a burgeoning interest in harnessing the therapeutic potential of PRP in dermatology, particularly in wound healing, vitiligo, scar revision, tissue regeneration, and skin rejuvenation. Additionally, research has explored its efficacy in addressing alopecia, offering promising avenues for managing hair loss.^4^, ^13^, ^14^, ^15^, ^16^, ^17^, ^18^, ^19^, ^20^, ^21^ The focus of this review will be on PRP for skin rejuvenation, an area of particular interest to the authors.

## 2 MATERIALS AND METHODS

Keywords including “platelet rich plasma,” “rejuvenation,” “skin aging,” “wrinkles,” were searched on Ovid, PubMed, MEDLINE databases for relevant studies published on PRP treatment. Some papers were further reviewed using objective endpoint measurements, a double‐blinding approach, control usage, randomization usage, and sample size. All studies were classified according to the Oxford Center for evidence‐based medicine evidence hierarchy. Subsequently, 11 studies met the criteria for review.

## 3 RESULTS

Of the 11 studies meeting the search criteria for PRP in skin rejuvenation, conducted between 2014 and 2021 and involving a total of 382 patients, three were split‐face trials, with only one randomized controlled, double‐blinded trial,^5^ and eight were prospective cohort open‐label studies. This study summarizes the findings from these eleven studies, analyzing their designs, evaluation parameters, and treatment outcomes.

A series of three treatment sessions using either autologous platelet‐rich plasma, hyaluronic acid, or a combination of autologous platelet‐rich plasma and hyaluronic acid were administered to 93 eligible patients in a randomized controlled prospective study conducted by Hersant et al.^22^ With a highly significant improvement in facial look and skin elasticity compared to either autologous platelet‐rich plasma or hyaluronic acid alone, the combination of autologous platelet‐rich plasma and hyaluronic acid appears to be a potential treatment for facial rejuvenation (Level 2b).

Elnehrawy et al.^23^ sought to evaluate the efficacy and safety of a single intradermal injection of autologous PRP for treating facial wrinkles and rejuvenating the face. This study comprised a total of 20 participants with various types of facial wrinkles. Clinical assessment tools such as the Wrinkle Severity Rating Scale (WSRS), Skin Homogeneity and Texture (SHnT) Scale, Physician Assessment Scale, and participant Satisfaction Scale were used to clinically evaluate each participant before and after receiving a single intradermal injection of PRP. Following 8 weeks of treatment, the mean value of WSRS decreased from 2.90 ± 0.91 before treatment to 2.10 ± 0.79. Particularly, younger patients with mild to moderate nasolabial fold wrinkles showed the most notable benefits. of seventeen subjects, fourteen had Fourteen of seventeen subjects with nasolabial folds demonstrated more than 25% improvement in their appearance. Side effects associated with PRP treatment were minimal to mild, and the procedure was well tolerated, with excellent tolerability noted. Fourteen out of 17 nasolabial fold wrinkles showed an improvement in appearance of more than 25%. The PRP procedure resulted in minimal to minor side effects and was well tolerated (Level 3b).

A prospective study was carried out by Yuksel et al.^24^ to determine the effects of the PRP application technique on human facial skin. On the face of ten healthy participants, three applications of autologous PRP were made at intervals of 2 weeks. A derma roller was used to apply it to the patient's forehead, malar region, and jaw, and a 27‐gauge injector was used to place it into the crow's feet creases. Prior to each PRP therapy and three months following the last PRP procedure, participants were asked to rate the general appearance, skin firmness‐sagging, wrinkle state, and pigmentation disorder of their own face on a scale of 0–5. The subjects’ faces were evaluated by themselves while simultaneously being evaluated by three separate dermatologists using the same five‐point scale. According to the grading scale used to compare the patients’ general look, skin firmness‐sagging, and wrinkle status before and after three PRP administrations, there was a statistically significant difference. However, in the dermatologists' opinion, there was only a statistically significant difference in the skin firmness‐sagging (Level 3b).

Eleven subjects were enrolled in the trial by Everts et al.^25^ and received three treatments with PRP. After 3 months, there was a significant reduction in brown spot counts and area (*P* < 0.05). A substantial decrease in wrinkle volume and count was observed (*P* < 0.05 for overall wrinkle look). Significant improvements were made to skin firmness parameters. After 169 days post‐therapy, skin redness in the nasolabial and malar areas had significantly decreased. At two months following the initial injection, a decrease in subepidermal low echogenic band thickness was seen, along with an increase in subepidermal low echogenic band density (*P* < 0.05 for both parameters), without changing subcutaneous fat thickness. At the 6‐month follow‐up, a self‐evaluation revealed an average satisfaction score of >90%. According to the authors, a series of three PRP injections. According to the authors’ findings, a series of three PRP injections led to significant skin renewal at a 6‐month follow‐up, which was supported by biometric measures and patient self‐assessment scores (Level 3b).

A total of 30 healthy females were enlisted by Du et al.^26^ for PRP therapy, and their signed informed consent was obtained. Each patient received a total of three autologous platelet‐rich plasma injections, spaced by 15 days. Using skin computed tomography, the effects of PRP injections were assessed. Hematoxylin and eosin and Masson's trichome staining were used to examine the distribution of the epidermal structure and dermal fibers. Reverse transcription quantitative polymerase chain reaction, western blotting, and immunofluorescence were used to find the expression of matrix metalloproteinase 1 (MMP 1), tyrosinase, fibrillin, and tropoelastin. The results of this study demonstrated that PRP treatment enhanced the participants’ skin quality. The in vitro study showed that platelet‐rich plasma therapy reduced photoaging by blocking UV B‐induced upregulation of MMP‐1 and tyrosinase and by causing UV B‐induced downregulation of tropoelastin and fibrillin expression. Overall, it was shown that PRP treatment reduced skin photoaging by controlling the production of MMP 1, tyrosinase, fibrillin, and tropoelastin [Level 3b].

Twelve patients were enlisted by Cameli et al.^27^ and had three PRP injection sessions spaced 1 month apart. Transepidermal water loss, corneometry, Cutometer, Visioscan, and Visioface were used to measure the clinical and instrumental outcomes before (T0) and one month (T1) after the conclusion of treatment. On samples of PRP and peripheral blood (PB), a flow cytometry characterization was completed. Skin texture improved as determined by clinical and patient evaluation. Skin capacitance, skin barrier function, skin smoothness parameters, and skin gross elasticity all saw significant improvements. Immunological differences between PB lymphocytes and PRP were not seen. All the PRP samples had a depleted neutrophil population and leukocyte population (mostly CD3+). This important study proved that skin biostimulation can be objectively improved by PRP low in leukocytes. Using a dependable separation technology and a low concentration of proinflammatory cells, flow cytometry revealed no variation between the PRP samples. Despite being a pilot study, it might be useful for future research on PRP cellularity (Level 3b).

To assess the effectiveness and safety of intradermal injection of PRP in the treatment of human face aging, Abuaf et al.^28^ conducted a prospective, single‐center, single‐dose, open‐label, non‐randomized controlled clinical trial on PRP. Twenty women between the ages of 40 and 49 (mean age: 43.65 ± 2.43 years) participated in the study. PRP was injected into the entire face and right infra‐auricular region. Injections of saline were made to the left infra‐auricular region. Prior to PRP treatment, 28 days after PRP treatment, and saline (control) treatment, histopathological tests were done. The pre‐treatment, control, and PRP‐treated areas’ mean optical densities (MODs) of collagen were all measured. They were, in order, 539 ± 93.2, 787 ± 134.15, and 1019 ± 178. When the MOD of PRP was compared to the MOD of pre‐treatment, the MOD of PRP showed an improvement of 89.05%. On the PRP side, the mean MOD of collagen fibers was unquestionably higher (*p* 0.001). The ratio of PRP to saline improvement was 1.93:1 (89.05%–46.01%). There were no negative side effects of note. The authors concluded that PRP enhances dermal collagen levels by skin needling (the mesotherapy technique known as “point by point”) as well as growth factors (Level 3b).

In the study by Ulusal et al.^29^ the aim was to support and supplement the existing PRP injection guidelines with data and discussion. PRP and hyaluronic acid (HA) were used to treat 94 female patients with varied degrees of face aging symptoms. 53.0 ± 5.6 was the mean age. The average injection was 3.6 ± 2.0. 0.5 cc of 3.5% hyaluronic acid, 0.5 cc of procaine, and portions of platelet‐poor and platelet‐rich plasma were combined before being injected with a 30G, 13‐mm needle into the deep dermis and hypodermis. Patients were asked to rate how happy they were with the sagging, color, and texture of their skin. Three impartial doctors and the patients themselves also evaluated the overall outcomes. The results underwent peer review, and associations between the number of injections and the degree of aesthetic scores were investigated. According to the patients’ before and after PRP administrations, there was a statistically significant difference in general appearance, skin firmness‐sagging, and skin texture. It was shown that there was a statistically significant association between the number of injections and overall pleasure. The authors concluded that the PRP and HA injections improved the facial skin in a clinically and statistically meaningful way over the control group. With more injections, the results became more striking (Level 3b).

In a study by Lee et al.,^30^ the effectiveness of a single PRP therapy performed using a straightforward preparation and application technique was evaluated along with patient satisfaction. For this investigation, 31 volunteers with ages ranging from 27 to 71 (median, 38; IQR 32–58) were enlisted. Six standardized locations were given four milliliters of PRP injections on either side of the face. Using the Wrinkle Severity Rating Scale (WSRS) and Global Aesthetic Improvement Scale (GAIS), independent physicians evaluated pretreatment and posttreatment images to determine the results. Only one patient's post‐treatment WSRS ratings improved; however, 14 patients' GAIS scores showed aesthetic improvement. The most common negative effects were pain (seven of 31;23.4%), tightness in the face (six of 31; 20.0%), and edema (six of 31; 20.0%). The study's authors concluded that addressing the signs of photodamage and skin aging with a straightforward PRP preparation approach delivers only marginal benefits (Level 3b)

Iranian researchers Banihashemi et al.^31^ set out to evaluate the effectiveness of pure PRP injections for facial rejuvenation. PRP was injected into 30 female subjects in a row over the course of two treatments, separated by 3 months. Comparisons between the pre‐and post‐improvement measurements of skin scans, before‐and‐after photographs by participants, therapeutic physicians, and blindly by a second dermatologist were used to perform evaluations. Patients reported moderate to excellent improvement in periorbital dark circles (47.8,60.9%), periorbital wrinkles (73.9%,78.3%), nasolabial fold (52.2%,56.6%), and skin rigidity (52.3%,60.9%) in the 3‐ and 6‐month follow‐ups, respectively. However, only periorbital dark circles (*P* value 0.031) showed statistical significance. According to a therapy physician's evaluation, there was moderate to good improvement in periorbital dark circles (47.9%, 74%), periorbital wrinkles (39.1%, 43.5%), and nasolabial folds (4.3%, 13.1%). Dark circle improvement (*p* < 0.05) and nasolabial fold improvement (*p* < 0.05) were statistically significant. By a second dermatologist, there was statistically significant improvement in periorbital dark circles (34.8%, 52.2%), periorbital wrinkles (26.1%, 34.8%), and nasolabial folds (4.4%, 13%). The strongest outcomes were shown in reducing periorbital wrinkles and dark circles, according to the authors, who concluded that face rejuvenation with PRP is a promising and noninvasive treatment (Level 3b).

A validated subjective scale, the FACE‐Q, along with an objective assessment using a Cutometer were used in open‐label prospective trial conducted by Hersant et al.^32^ to determine the clinical value of combining PRP and HA (PRP‐HA) effectors, which have synergistic effects on skin firmness and elasticity. The study recruited 31 patients in all, with a mean age of 51.8 plus or minus 8.5 years. When comparing FACE‐Q scores, it was found that there had been a substantial improvement after six months (44.3 ± 1.9 at baseline compared to 52 ± 3.17 at six months, *p* < 0.05). Similar improvements were seen in biophysical parameters for R5 (*p* < 0.05) compared to the starting point. There were no documented severe negative events (Level 3b).

The most common treatment protocol was PRP injections given three times with a 2−3‐week interval in between. The centrifuge settings used to prepare the PRP varied among the studies. Some studies used a single spin, while others used a double spin setting for the first and second spins. The reported duration of the first spin varied from 5–10 min, and the second spin varied from 3–10 min.

The physical assessments showed that PRP treatment improved skin wrinkles, tone, elasticity, skin turgor, and epidermal and dermal thickness (*p* < 0.05) pores, overall facial appearance (*p* < 0.05).^22^, ^23^, ^24^ The improvement in brown spots and counts was also significant (*p* < 0.05).^25^

Biometric assessments using the VISIA® skin analysis system demonstrated significant improvement in facial wrinkles, texture, skin homogeneity, elasticity, and firmness (*p* < 0.05).^25^

The cutometer and skin CT showed a significant improvement in skin elasticity and firmness following PRP (*p* < 0.05).^26^ One study reported a synergetic effect when HA was added to PRP. A negative correlation between the skin elasticity and body mass index was observed, together with an age effect to which patients in their 50s and 60s showed the greatest improvement in skin tightness following PRP treatment.^22^

PRP treatment also improved skin smoothness, gross elasticity, capacitance, and barrier function, as quantitatively measured by Cutometer, Visioface, Visioscan, corneometry, water loss, and trans‐epidermal water loss evaluations. Flow cytometry showed reproducibility in PRP samples and a low content in pro‐inflammatory cells.^27^

In vivo studies established that PRP treatment increased the density of dermal collagen (*p* < 0.05).^11^

There was evidence to support PRP treatment may protect human skin from UV‐induced damage by regenerating gene expressions of MMP‐1, tyrosinase, fibrillin, and tropoelastin, observable at both mRNA and protein levels.^26^

Overall, this study suggests that PRP monotherapy is an effective treatment for skin rejuvenation, with improvements observed in various skin parameters. However, further research is needed to determine the optimal treatment protocols and centrifuge settings for PRP preparation.

All eleven studies that evaluated patient‐reported outcomes noted satisfaction and cosmetic improvements. There was a corresponding improvement in patients’ satisfaction (GAIS), an average satisfaction score of >90% at the 6‐month follow‐up, and an improvement in facial appearances (FACE‐Q scores) (*p* < 0.05).^24^, ^27^, ^30^ Overall, the results suggest that PRP can be an effective treatment for facial skin rejuvenation, significant improvements in skin quality, texture, and wrinkles, as well as improvements in hyperpigmentation, skin firmness, and redness. However, caution should be exercised when interpreting the results due to the differences in PRP preparation methods and patient factors such as age and skin quality. The optimal protocol for PRP preparation and use may also vary depending on specific centrifugation settings, duration, and additional treatments such as hyaluronic acid.

In terms of safety, PRP monotherapy is considered safe, with no severe adverse events reported. However, common side effects such as bleeding, bruising, infection, and pain can occur, and infection and contamination are rare but possible if proper aseptic technique is not followed. Although the risks of immune allergic reactions and hypersensitivity are rare, they can occur if the patient is allergic to any component of PRP.

Nevertheless, complications can occur. In other PRP case series, seven cases of unilateral vision loss or impairment after PRP injection had been reported outside of this review. Unfortunately, the details of PRP composition, preparation, and injection techniques were not available.^33^, ^34^, ^35^ Similar to filler‐induced vascular occlusion (FIVO), the glabellar remained the most vulnerable area because of its limited collateral circulation from supratrochlear and supraorbital arteries. Visible loss or impairment is rare but has a grave prognosis. Only one of the seven patients completely recovered vision after three months. There should be a high suspicion of occlusions and start treatment without delay to reverse the obstruction and minimize ocular and tissue damage.^36^, ^37^

## 4 DISCUSSION

Careful patient selection is critical and excludes those with active infection and systemic use of corticosteroid or non‐steroid anti‐inflammatory two weeks before the procedure. The use of PRP is also relatively contraindicated in patients with the following conditions: anti‐coagulant therapy, cancer, oral contraceptive use, or a history of deep vein thrombosis.^38^ We must also be careful in preventing cross‐contamination, mislabeling, or misidentifying of a patient's blood during PRP preparation. PRP contains a variety of cytokines and growth factors that promote wound healing and tissue regeneration, including platelet‐derived growth factor, transforming growth factor‐beta, vascular endothelial growth factor, and insulin‐like growth factor‐1. These growth factors stimulate collagen synthesis, angiogenesis, and possess anti‐inflammatory and immune‐modulatory properties. Clinical trials have demonstrated that PRP can improve skin texture, tone, and elasticity, while also reducing the appearance of wrinkles, fine lines, and pigmentation changes.^5^, ^39^

PRP can be prepared by either a one or two‐step centrifugation process that concentrates platelets from the patient's blood. However, factors such as centrifugation speed, time, and temperature, as well as the use of different anticoagulants, can affect the final composition of PRP.^40^ These variations in PRP preparation can lead to differences in the concentration of platelets, white blood cells, and growth factors, which can affect PRP's therapeutic efficacy.^41^ Therefore, establishing standardized protocols for PRP preparation is essential to ensure consistent quality and efficacy of the final product.

Our literature review of PRP for skin rejuvenation highlights a potential limitation in the heterogeneity of protocols used for PRP preparation in the studies included. The differences in PRP kit sets and preparation protocols can impact the final composition of PRP and limit the conclusions that can be drawn from the studies. In response to this limitation, we have carefully considered the PRP preparation methods used in each of the studies included in our review. Despite the observed similarity in platelet concentration and capture efficiency, the differences in other aspects of PRP preparation protocols may still impact the outcomes observed. Therefore, we advise caution when interpreting the results of studies with different PRP preparation protocols.

## 5 CONCLUSION

Based on our review, it can be concluded that PRP monotherapy positively impacts skin quality and texture, wrinkles, and hyperpigmentation. The studies demonstrate significant improvements in skin quality and texture, including a reduction in brown spot counts, wrinkle count, and volume, improvement in skin firmness and redness, and a significant increase in the mean optical densities of collagen. Patient‐reported outcomes and satisfaction rates are consistently high at 6‐month follow‐ups. PRP monotherapy is safe, with few adverse effects reported, such as pain, discomfort, and bruising at the injection site. However, complications such as infection, contamination, and occlusion can occur. The studies’ outcomes should be interpreted cautiously due to the small sample sizes and non‐randomized study designs in some studies. Overall, PRP monotherapy is a safe and effective treatment for facial skin rejuvenation, with high patient satisfaction rates, and promising results.

## CONFLICT OF INTEREST STATEMENT

I acknowledge that I have considered the conflict of interest statement included in the “Author Guidelines.” I hereby certify that, to the best of my knowledge, that no aspect of my current personal or professional situation might reasonably be expected to significantly affect my views on the subject I am presenting.

## Data Availability

The data that support the findings of this study are available from the corresponding author upon reasonable request.
